# The effect of topical ocular moxifloxacin on conjunctival and nasal mucosal flora

**DOI:** 10.1038/s41598-021-93233-5

**Published:** 2021-07-02

**Authors:** Ali Riza Cenk Celebi, Ozlem Onerci Celebi

**Affiliations:** 1 Department of Ophthalmology, School of Medicine, Atakent Education and Research Hospital, Acibadem University, Turgut Ozal Blvd. No:16, Kucukcekmece, 34303 Istanbul, Turkey; 2 Department of Otorhinolaryngology, Eregli State Hospital, Toros Mah. Yurt Cad. No: 13, Eregli, 42000 Konya, Turkey

**Keywords:** Antimicrobials, Microbiology, Clinical microbiology

## Abstract

To determine the short-term effect of topically administered ocular moxifloxacin on conjunctival and nasal bacterial mucosal flora. The study included 20 patients with newly diagnosed age-related macular degeneration. Each patient’s diseased eye was selected as the treatment eye and the fellow eye was selected as the control eye. All treatment eyes constituted the treatment group and all controls eyes constituted the control group. All patients received intravitreal injection of ranibizumab. Cultures were obtained from the inferior conjunctival fornix and the nostrils in all patients. Patients were instructed to administer moxifloxacin eye drops to the treatment eye 4 times daily for 1 week. The patients were instructed to come for a follow-up exam 1 week post intravitreal injection. The bacterial culture positivity rate and the bacteria isolated from the conjunctiva and nostrils were recorded in the 2 groups before and after use of topical ocular moxifloxacin. Mean age of the patients (12 female and 8 male) was 64.9 years. Before use of topical ocular moxifloxacin the conjunctival and nasal culture positivity rates in the treatment group were both 100%, versus 90% and 95%, respectively, in the control group. At the follow-up exam the conjunctival and nasal mucosa culture positivity rates in the treatment group decreased to 20% (4/20) and 30% (6/20), respectively (P < 0.001), versus 85% (17/20) and 80% (16/20), respectively, in the control group (P = 0.68 and P = 0.72 for conjunctival and nasal). This is the first study to show that moxifloxacin applied to the ocular surface topically has a significant effect on nasal flora. Daily administration of topical ocular moxifloxacin for 1 week significantly reduces the nasal bacterial flora in addition to conjunctival flora.

## Introduction

Antibacterial ophthalmic solutions containing fluoroquinolones are widely used for perioperative disinfection because they effectively eradicate bacteria before ophthalmic surgery^1^. Newer fourth generation fluoroquinolones (moxifloxacin and gatifloxacin) are gaining in popularity, as they are readily available, exhibit a wide range of action against gram-positive and numerous gram-negative microbes, have a low resistance rate and great solubility, and are less toxic^2^.

Intravitreal injection of various anti-VEGF agent for the treatment of age-related macular degeneration (AMD) is among the fastest growing ophthalmological procedures. The frequency of endophthalmitis after intravitreal injection is thought to be about 0.2% per injection^3^. Guidelines for limiting the occurrence of endophthalmitis due to intravitreal injections were published in 2004, but did not include the use of antibiotics prior to injection^3^. Nonetheless, effective antibiotics are still generally utilized in ophthalmological practice despite a lack of evidence of their efficacy^4^.

It is well known that the nasopharynx is exposed to anti-microbials following topical instillation in the eyes^5,6^. The topical medications used on ocular surface can pass through these anatomical pathways (the nasolacrimal duct) and can have a significant effect on nasal flora as they are connected. Roughly 40% of a standard 50-μL eye drop straightforwardly enters the highly vascular tear drainage system, and is then absorbed by the nasopharyngeal mucosa^5^. As the nasal cavity and the eyes are connected to each other via the nasolacrimal ductus, alongside their proximity, infection in either can convey the risk of transmission to the other^6^. This indicates that there could be transmission of a microbiological between the nose and the eyes. Besides, the ocular surface and the nasal area share similar bacteriological flora. Durmaz et al.^6^ stated that the most common microorganisms in ocular and nasal cultures were similar; CNS, *Staphylococcus* species, and Diphtheroid spp.

The Antibiotic Resistance of Conjunctiva and Nasopharynx Evaluation (ARCANE) prospective controlled longitudinal study aimed to determine the effect of anti-microbials on the resistance status of conjunctival and nasopharyngeal flora after rehashed administration of ocular anti-microbials in patients that underwent intravitreal injections^7^. The study showed that rehashed utilization of fluoroquinolone ophthalmic anti-microbials quickly increased the amount of resistant conjunctival strains of coagulase-negative staphylococci (CNS)^7^, which are microorganisms that potentially colonize in the ocular and nasal environment. Nonetheless, the effect of topically administered ophthalmic anti-microbials on conjunctival and nasal flora in intravitreal injection-naive AMD patients was not assessed. To the best of our knowledge no recently published study in the English-language literature has investigated the effect of topically administered ocular anti-microbials on conjunctival and nasal flora in intravitreal injection-naive AMD patients. The present study, therefore, aimed to determine the effect of ocular moxifloxacin on conjunctival and nasal bacterial mucosal flora in intravitreal injection-naive AMD patients.

## Materials and methods

This prospective study included all patients aged > 50 years that were diagnosed with choroidal neovascularization due to AMD in 1 eye only and underwent intravitreal injection of ranibizumab. Exclusion criteria were a history of intraocular injection in either eye, ocular surgery, and long-term use of any ophthalmic medications, contact lens wear, current utilization of ophthalmic drugs in either eye or ocular disease during the previous 3 months, and use of oral systemic anti-infective agents during the previous month.

All patients had an intact nasolacrimal passage that was confirmed via nasolacrimal irrigation. After the diagnosis of neovascular AMD was established, a culture of the conjunctival surface was obtained prior to intravitreal injection of ranibizumab. When the study began cultures of the inferior fornix of each treatment eye and control (contralateral) eye were performed prior to administration of proparacaine and 5% povidone iodine in the treatment eyes, which was performed with care to ensure that the administration swab did not make contact with the eyelids or eyelashes. Nasal cultures were obtained bilaterally, by gently rolling the swabs around the nasal mucosa. Both the conjunctival and nasal culture swabs were inoculated onto sheep blood agar and chocolate agar plates, and were then incubated at 37 °C for 3 days. Even a single colony was considered as a positive culture result for both conjunctival^8^ and nasal cultures^9^. Isolated microorganisms were identified using standard microbiologic methods, including Gram stain, coagulase test, catalase test, and oxidase test. Patients with positive conjunctival and nasal cavity cultures bilaterally prior to the start of the study were included, so as to study the impact of effective ocular anti-microbials. Sensitivity analysis for moxifloxacin was obtained in each patient prior to treatment. Patients whose culture results showed organisms that were resistant to moxifloxacin were excluded from the study.

A standard procedure was adhered to for all intravitreal injections. All treatment eyes were prepared with 5% povidone-iodine before intravitreal injection and each treated eye was instilled with a drop of moxifloxacin immediately thereafter. Patients were instructed to instill anti-microbial drops in the treated eye 4 times daily for 7 days. The patients were also instructed to come for a follow-up exam 1 week post injection during which time conjunctival and nasal cultures were again obtained using the same methods described above. The bacterial culture-positivity rate and the bacterial species noted were also recorded at this time. Decrease in rate of positive cultures and the increase of negative cultures after the antibiotic treatment were accepted as a reduction in growth. The study protocol was approved the Acibadem University School of Medicine Ethics Committee and was conducted in accordance with the Declaration of Helsinki for research including humans. Written informed consent was provided by all the participants.

### Statistical analysis

Statistical analysis was performed using IBM SPSS Statistics for Windows v.22. (IBM Corp., Armonk, NY). Descriptive measurements, including mean age of the patients and culture positivity rates, were made. The rates of culture positivity in the treatment and control eyes both before and after prophylactic antibiotic therapy were compared via the McNemar test. Group comparisons (treatment eyes versus control eyes) of ocular and nasal culture positivity was performed using Fisher’s exact test. The level of statistical significance was set at P < 0.05.

## Results

The study included 20 patients (12 female and 8 male) with a mean age of 64.9 years.

Patients that had positive culture results in the treatment eye and corresponding nasal mucosa, regardless of microorganism, were included; therefore, the culture positivity rate in the treatment eyes and corresponding nasal mucosa was 100%.

In the control group the conjunctival and corresponding nasal culture positivity rates at the beginning of the study were 90% and 95%, respectively.

Prior to treatment, differences in the conjunctival and nasal culture positivity rates between the treatment and control eyes were not significant (P = 0.78 and P = 0.82 for conjunctival and nasal culture rates, respectively; Fisher’s exact test).

However, after 1 week of moxifloxacin treatment, the conjunctival and nasal culture positivity rates between the treatment and control groups differed significantly (P < 0.001 for both conjunctival and nasal culture rates; Fisher’s exact test).

### Conjunctival flora characteristics

The bacterial species cultured from the treatment and control eyes, were CNS, *Staphylococcus aureus*, and *Corynebacterium* species. All reported species were moxifloxacin-sensitive according to the sensitivity testing.

The distribution of the isolates from the conjunctival culture prior to moxifloxacin antibiotic therapy are summarized in the Table 1.

**Table 1:** The distribution of isolates from conjunctival and nasal cultures prior to and after antibiotic therapy in the treatment and control eyes.

Microorganism type	Pre-treatment				Post-treatment			
	Treatment eyes % (n)		Control eyes % (n)		Treatment eyes % (n)		Control eyes % (n)	
	Conjunctival culture	Nasal culture	Conjunctival culture	Nasal culture	Conjunctival culture	Nasal culture	Conjunctival culture	Nasal culture
CNS	60% (12)	60% (12)	55% (11)	50% (10)	10% (2)	20% (4)	50% (10)	45% (9)
*S. aureus*	25% (5)	20% (4)	20% (4)	20% (4)	5% (1)	5% (1)	20% (4)	20% (4)
Corynebacterium	15% (3)	20% (4)	20% (4)	20% (4)	5% (1)	5% (1)	15% (3)	15% (3)
Total	100% (20)	100% (20)	95% (19)	90% (18)	20% (4)	30% (6)	85% (17)	80% (16)

The numbers show the percent and the number of positive cultures for each group.

After 1 week of topical ocular moxifloxacin treatment only 4 of 20 isolates (20%) were cultured from the conjunctiva in the *treatment group* (decrease from 100%), of which 2 were CNS, 1 was *Staphylococcus aureus*, and 1 was *Corynebacterium*, this total amount of decrease of isolates was significant (P < 0.001; McNemar test). Reported species after the treatment period were also moxifloxacin-sensitive according to the sensitivity testing.

After 1 week of topical ocular moxifloxacin treatment 17 of 20 (85%) isolates were cultured from the conjunctiva in the *control group* (decrease from 90%), of which 10 were CNS (11 prior to treatment period), 4 were *Staphylococcus aureus* (4 prior to treatment period) and 3 were *Corynebacterium* (4 prior to treatment period) however, these decreases in the conjunctival culture positivity rates were not significant (P = 0.68; McNemar test). Reported species both before and after the treatment period in control eyes were also moxifloxacin-sensitive according to the sensitivity testing.

The distribution of the isolates from the conjunctival cultures after antibiotic therapy in both the treatment and control groups are summarized in the Table 1.

### Nasal flora characteristics

The bacterial species cultured from the side of treated eye’s and control eye’s nasal cavities were CNS, *Staphylococcus aureus*, and *Corynebacterium* species. All reported species were moxifloxacin-sensitive according to the sensitivity testing.

The distribution of the isolates from the conjunctival culture prior to moxifloxacin antibiotic therapy are summarized in the Table 1.

After 1 week of topical ocular moxifloxacin treatment only 6 of 20 (30%) isolates were cultured from nasal mucosa in the *treatment group* (decrease from 100%), of which 4 were CNS, 1 was *Staphylococcus aureus*, and 1 was *Corynebacterium*, this total amount of decrease of isolates was significant (P < 0.001; McNemar test). Reported species after the treatment period were also moxifloxacin-sensitive according to the sensitivity testing.

After 1 week of topical ocular moxifloxacin treatment 16 of 20 (80%) isolates were cultured from the nasal mucosa of the *control group* (decrease from 95%), of which 9 were CNS, (10 prior to treatment period), 4 were *Staphylococcus aureus* (4 prior to treatment period) and 3 were *Corynebacterium* (4 prior to treatment period); however, these decreases in the nasal culture positivity rates were not significant (P = 0.72; McNemar test). Reported species both before and after the treatment period in the nasal mucosa of the control eyes were also moxifloxacin-sensitive according to the sensitivity testing.

The distribution of the isolates from the nasal cultures after antibiotic therapy in both the treatment and control groups are summarized in the Table 1.

## Discussion

The present findings show that the use of ocular application of moxifloxacin significantly alters the distribution of nasal bacterial flora in addition to conjunctival flora. In other words, one of the most frequently used topical antibiotics used for prophylaxis of endophthalmitis after intravitreal injection has a significant antibacterial effect on microorganisms normally present in the nasal bacterial flora. Moxifloxacin has a rapid onset of action for reducing the commensal bacterial load in both the conjunctival surface and nasal mucosa flora.

Microorganism colonization of the ocular surface and encompassing tissues is a powerful cycle and it is widely acknowledged that most people harbor a few microbes on their ocular surface^10^. Bacteria cultured from the conjunctival sac are typically similar to those in the upper respiratory tract. The most common organisms cultured from the conjunctiva are *Staphylococcus* species and *Corynebacterium* species^11^. It is thought that these typical ocular microscopic organisms serve a defensive function under most conditions via direct suppression of colonization of more pathogenic species^12^. It was reported that *S. epidermidis* may have a probiotic function by preventing colonization of the more dangerous *S. aureus* strain^13^. This phenomenon—known as ‘competitive exclusion’—has been studied in other anatomic sites including the nares^14^. So alteration of the nasal mucosa might increase the tendency of an individual infected by more severe pathogens like MRSA.

As the nasal cavity and the eyes are connected to each other by the nasolacrimal system, contamination in one can convey the risk of contamination in the other^6^. Similar bacteriological flora between the nose and the eye is also the result due to close proximity with the presence of anatomical structures like nasolacrimal duct. In our study we found similar bacteriological profile in our both conjunctival and nasal flora (CNS, *Staphylococcus* species, and *Diphtheroid* spp.), consistent with the literature^6^.

Topical ocular antibiotic administration for the prevention of endophthalmitis prior to and after intraocular surgery has become routine practice^15^. However, the change of the resistance profiles of antibacterial solutions over time should be discussed. Nejima et al.^16^ found that susceptible CNS strains rate decreased from 73.6 to 20.2% after 3 day of quinolone administration after cataract surgery, suggesting an increase in resistance. Even after cessation of the drop, the susceptible strains rate only increased to 38.5% at 3 months follow up. This was explained with the changes of the MIC levels of quinolones isolated from the normal conjunctival CNS bacterial flora. Following this result, we can conclude that even short-term ocular administration of high-concentration antimicrobial ophthalmic solution can change and induce CNS resistance. In addition to CNS, resistance to other conjunctival bacterial flora microorganisms like Corynbacterium strains have also been reported. Deguchi et al.^17^ published a study in 2018 and investigated trends of resistance to bacterial antibiotics compared with 10-years previous for common ocular surface microorganisms (CNS, *Staphylococcus* species, and *Diphtheroid* spp.). It was reported that the prevalence of quinolone-resistant Corynebacterium strains has not changed in the past 10 years. However, a recent report by Hoshi et al.^18^ studied nasal flora in addition to conjunctival flora and showed an increased quinolone resistance in both nasal and conjunctival Corynebacterium profiles. A more recent review by Aoki et al.^19^ which also studied nasal flora in addition to conjunctival flora pointed out that Corynebacterium species present on the ocular surface were resistant to quinolones, whereas those in the nasal cavity were more susceptible. That difference was explained by the increased exposure to quinolone antibiotics on the conjunctival surface. Although antibiotic resistance and the amount of bacterial load for conjunctival and nasal flora has been previously reported as mentioned above^18,19^, alteration of conjunctival and correspondent nasal bacterial flora after short-term topical administration of ocular antibiotics has not been previously studied. As we only included the patients that were quinolone (moxifloxacin) sensitive, we were unable to obtain a percentage of ocular and nasal flora that were quinolone resistant and we hope that this can be topic of a further study.

The most common organisms in the present study were CNS, as reported earlier^20^. CNS are responsible for roughly 70% of postoperative endophthalmitis cases^21^ and eliminating this pathogen is of a paramount importance; therefore, anti-microbials are utilized as often as possible both prior to and after each intravitreal injection. Reviews have shown that 40% of retina experts use anti-infection agents before intravitreal injections and that 86% of retina experts use anti-microbials after intravitreal injection^22^.

Use of topical fourth generation fluoroquinolone for 3 days significantly decreases conjunctival bacterial growth^23^; in the present study use of topical fourth generation fluoroquinolone also significantly reduced conjunctival bacterial growth; however, there are no data available on nasal bacterial flora alteration after short-term use of topical administration of ocular moxifloxacin. In the present study there was a significant decrease in bacterial growth on nasal mucosal flora after 1 week of fourth generation fluoroquinolone use (from a 100 to 30% bacterial culture positivity rate).

*Staphylococcus epidermidis* in the conjunctival sac was also noted to play a significant role in the nasal cavity^24^. In the present study CNS, *Corynebacterium* species, and *S. aureus* were the most common microorganisms in the nasal cavity, as previously reported^25,26^. These organisms are a frequent cause of bacteriemia and have been implicated as the source of surgical site infections in various surgical populations^27^. Kimura et al.^28^ observed that there is a significant relationship between ocular surface colonization and nasal carriage of methicillin-resistant *S. aureus* (MRSA) strains in their cohort. Endophthalmitis is an important complication of intraocular ophthalmic procedures and Speaker et al.^29^ reported that micro-organisms obtained from the vitreous body were genetically different than those obtained from the nose in patients with endophthalmitis. Moreover, the nasal mucosa was noted to be a store of micro-organisms for the conjunctiva and nasolacrimal passage. Alexandrou et al.^30^ observed a decrease in preoperative conjunctival bacterial flora following nasal use of mupirocin ointment.

Decreasing or changing the distribution of the typical nasal commensal bacterial flora can promote the recurrence of pathogenic nasal MRSA strains, which is a typical cause of nosocomial diseases in clinical settings^31,32^. Human studies have affirmed that nasal commensal microbials, for example, *S. epidermidis*, compete with rival *S. aureus* in the same environment^31,32^; therefore, decreasing the quantity of colonized commensal *S. epidermidis* in the nasal flora with anti-bacterial drugs may result in an increase in nasal MRSA colonization. One study reported that the ordinary nasal flora microbial *S. epidermidis* fundamentally limits the infectivity of various influenza strains; as such, decreasing the nasal *S. epidermidis* population could result in flu flare-ups^33^.

The present study has a few limitations, including a small sample and single-center design. Larger scale, longer term, and multicenter studies are likely to yield more robust data. Another limitation of this study is that only aerobic cultures were prepared from the specimens; nevertheless, obligate anaerobes could behave differently after short-term use of topical moxifloxacin. The present study aimed to determine the short-term effect of topical ocular moxifloxacin on nasal mucosal flora, but it would be beneficial to determine if the observed antibacterial effect on the nasal mucosal flora is long lasting, even after prolonged cessation of antibiotic use. One other limitation of our study is related to detailed investigation of antibiotic resistance profile on both conjunctival and nasal floras. However, as the primary medication we used was moxifloxacin, we primarily investigated the moxifloxacin resistance profile and excluded the patients who were resistant to moxifloxacin. Obtaining a detailed analysis of antibiotic resistance could have been helpful but was not the primary aim of our study as we only included the moxifloxacin sensitive patients. Another limitation of this study is the lack of microbiota analysis with resolution to species or strain level, which is currently underway.

In conclusion, this is the first study to show that moxifloxacin applied to the ocular surface topically has a significant effect on nasal flora. Daily administration of topical ocular moxifloxacin for 1 week significantly reduces the nasal bacterial flora in addition to conjunctival flora. The impact of topical ocular antibiotics on nasal flora should be well understood and should be kept in mind when prescribing these medications.
